# Cognitive load and autonomic response patterns under negative priming demand in depersonalization‐derealization disorder

**DOI:** 10.1111/ejn.13183

**Published:** 2016-02-15

**Authors:** Erwin Lemche, Mauricio Sierra‐Siegert, Anthony S. David, Mary L. Phillips, David Gasston, Steven C.R. Williams, Vincent P. Giampietro

**Affiliations:** ^1^ Institute of Psychiatry, Psychology & NeuroscienceKing's College School of Medicine and DentistryDepartment of Psychosis StudiesSection of Cognitive NeuropsychiatryBox PO69, Office 7.05, Basket 32De Crespigny ParkLondon SE5 8AFUK; ^2^ Western Psychiatric Institute and ClinicUniversity of Pittsburgh School of MedicinePittsburghPAUSA; ^3^ Department of NeuroimagingInstitute of Psychiatry, Psychology and NeuroscienceDe Crespigny ParkLondonUK

**Keywords:** correlation image analysis, effective connectivity, electrodermal activity, evoked haemodynamic responses, functional magnetic resonance imaging, Stroop word–colour interference

## Abstract

Previous studies have yielded evidence for cognitive processing abnormalities and alterations of autonomic functioning in depersonalization‐derealization disorder (DPRD). However, multimodal neuroimaging and psychophysiology studies have not yet been conducted to test for functional and effective connectivity under cognitive stress in patients with DPRD. DPRD and non‐referred control subjects underwent a combined Stroop/negative priming task, and the neural correlates of Stroop interference effect, negative priming effect, error rates, cognitive load span and average amplitude of skin conductance responses were ascertained for both groups. Evoked haemodynamic responses for basic Stroop/negative priming activations were compared. For basic Stroop to neutral contrast, patients with DPRD differed in the location (inferior vs. superior lobule) of the parietal region involved, but showed similar activations in the left frontal region. In addition, patients with DPRD also co‐activated the dorsomedial prefrontal cortex (*BA*9) and posterior cingulate cortex (*BA*31), which were also found to be the main between‐group difference regions. These regions furthermore showed connectivity with frequency of depersonalization states. Evoked haemodynamic responses drawn from regions of interest indicated significant between‐group differences in 30–40% of time points. Brain‐behaviour correlations differed mainly in laterality, yet only slightly in regions. A reversal of autonomic patterning became evident in patients with DPRD for cognitive load spans, indicating less effective arousal suppression under cognitive stress – patients with DPRD showed positive associations of cognitive load with autonomic responses, whereas controls exhibit respective inverse association. Overall, the results of the present study show only minor executive cognitive peculiarities, but further support the notion of abnormalities in autonomic functioning in patients with DPRD.

## Introduction

To maintain cognitive focus in concentration‐demanding tasks requires the ability to suppress perceptually competing influences or memory traces of preceding stimuli known as priming. Directing attention therefore requires the involvement of working memory, and the utilization of its specific capacities under cognitive load. Processing of memory content in priming also implies working memory being occupied. Classical research in information processing during problem solving in the context of goal attainment has revealed that short‐term memory acts in the service of executive control. If no previous schematic knowledge is available, perceived information is divided into amounts that fit into working memory capacity (Miller, 1956; Sweller, 1988). According to classical cognitive load theory, these amounts refer to short‐term memory and index individual differences in processing capacities. Higher cognitive loads cause psychophysiological stress, as attention requires cognitive selection, and this effort elicits autonomic arousal (Posner, 1975).

Depersonalization states are transient alienation symptoms frequently observed in prodromal stages of psychosis, posttraumatic stress, in personality, anxiety and depressive disorders, as well as in parts of the dissociative spectrum. Because abnormalities in memory functioning had been described, probing of attention and memory is of particular relevance also in persistent depersonalization‐derealization disorder (DPRD). Only few cognitive experiments have, however, been undertaken to examine the brain mechanisms underlying DPRD (Guralnik *et al*., 2000, 2007; Stein & Simeon, 2009). Specific abnormal relations of stress and depersonalization have been postulated (Stein & Simeon, 2009), and a ‘reversal of normal patterns of autonomic functioning’ was hypothesized by Phillips & Sierra (2003). The rationale for the present study was to elucidate possible attentional and mnestic alterations commonly implicated in the dissociative spectrum, as well as concurrent autonomic responding.

Recent meta‐analyses (Derrfuss *et al*., 2004, 2005; Neumann *et al*., 2005) have identified the mid‐dorsolateral prefrontal cortex and a posterior‐lateral prefrontal region at the intersection of Brodmann areas (BAs) 6, 8 and 9 as common brain regions involved in Stroop and other task‐switching experiments. This region, termed the inferior frontal junction, has been characterized to be cytoarchitectonically different from premotor, prefrontal and eye‐field regions (Brass *et al*., 2005; Amiez & Petrides, 2009; Derrfuss *et al*., 2012). Its functional connectivity network encompasses regions in ventrolateral and dorsolateral prefrontal as well as medial parietal regions (Sundermann & Pfleiderer, 2012). For the negative priming effect (NPE), Steel *et al*. (2001) demonstrated superior to inferior parietal (BAs 5,7, 40), superior, middle and inferior frontal (BAs 6, 8, 9, 45, 46), as well as occipital activations. To the authors’ knowledge, cognitive load had never previously been investigated in neuroimaging studies.

In the present study, multimodal neuroimaging of patients with DPRD and healthy controls was conducted by performing a combined negative priming and Stroop paradigm (negative priming Stroop) in two experimental conditions with known neural response properties, including derivation of sympathetic responses. A classical Stroop test without congruent condition was used, in order to avoid introducing a certain positive correlation into cognitive control processes (Dishon‐Berkovits & Algom, 2000). Previous behavioural experiments in DPRD (Guralnik *et al*., 2000) led to the assumption of no severe group differences in Stroop interference effect (SIE), NPE and cognitive load (hypothesis i). Regarding SIE, it was expected to find activation in the inferior frontal junction and parietal regions for normal controls (hypothesis ii). For the NPE, similar activations as described by Steel (see above) were expected, due to task similarity (hypothesis iii). For cognitive load, activations in regions where delay‐active neurons have been discovered were expected: prefrontal, posterior parietal and striatal structures (Fuster, 1973; Ashby *et al*., 2005; hypothesis iv). Following recent skin conductance findings on responses in cognitive tasks (Zhang *et al*., 2012), in which the supplementary motor area (SMA) regions were commonly found to be involved in several between‐condition contrasts, these same regions were expected to be the main brain‐behaviour correlation region in non‐referred control (NC) subjects (hypothesis v). For patients with DPRD, different brain‐behaviour correlation regions were expected, as it is well accepted that physiological stress impairs working memory performance (Arnsten, 1998, 2009) in the prefrontal cortex (hypothesis vi).

## Materials and methods

### Participants

Volunteers in the experiments were 12 healthy control subjects and nine patients with DPRD. The study was conducted in compliance with the Helsinki Declaration. All procedures had been endorsed by the local Research Ethics Sub‐Committee for the Institute of Psychiatry, Psychology & Neuroscience (RESC 141‐00). All participants signed informed consent and received monetary compensation for their time commitment. Primary‐diagnosis DPRD patients (mean age 36.11 ± 2.34 years; education level 2.22 ± 0.14 with 2 = junior college level; four females) from the Maudsley Hospital, London, UK, and NC subjects (mean age 27.25 ± 1.95 years; education level 2.58 ± 2.02; five females) participated in the experiments. At the time of investigation, patients were treated in a specialized clinic (ASD and MLP) for this diagnosis. All patients were co‐diagnosed with primary DPRD according to DSM‐5 criteria by a psychiatrist not involved in the study. Patients with DPRD were separately invited to participate in the study by the experimenter (EL), who was blind to all medical records. All patients exceeded the clinical cut‐off level of > 70 on the Cambridge Depersonalization Scale item version total scale discriminative for DPRD (Sierra & Berrios, 2000; 175.77 ± 12.31; Appendix S1).

### Behavioural paradigm

Subjects completed an experiment combining classical Stroop (Stroop, 1935; Jensen & Rohwer, 1966; MacLeod, 1991) and negative priming (Dalrymple‐Alford & Budayer, 1966; Tipper, 1985; Tipper & Cranston, 1985; MacLeod & MacDonald, 2000) tasks in a two‐condition version: a neutral control condition; and a Stroop/negative priming active probe condition (Steel *et al*., 2001). Each condition consisted of five alternating blocks of eight trials each. To acquaint subjects with the task demand, the initial block had three additional training trials (so 11 trials in total), which were later discarded (for computation of the SIE, NPE and average response‐delay spans (ARDS) for cognitive load, see Appendix S1).

### Psychophysiological recording

Electrodermal activity (EDA) measures were recorded online from within the MR scanner using the methodology described previously (Lemche *et al*., 2006). Applying threshold criteria of 0.01 μSiemens, EDA was analysed in whole epochs for each of the presentation blocks in each subject using the *SC‐ANALYZE* inhouse software (Centre for Neuroimaging Sciences, IOPPN, King's College London, UK). Following standard procedures, amplitude of skin conductance response (ASCR) was determined as the largest fluctuation (skin conductance response, SCR) of each epoch in μSiemens, and averaged for each condition and for each subject.

### Functional magnetic resonance imaging (fMRI) data acquisition

Imaging data were acquired using a neurovascular 1.5T GE scanner (General Electric, Milwaukee, WI, USA). Both structural and functional scans were acquired during the same session. High‐resolution structural images (43 slices), providing whole brain coverage [thickness: 3 mm; inter‐slice gap: 0.3 mm; planes parallel to the intercommissural (AC‐PC) line], were acquired using an inversion‐recovery EPI (T_*E*_ 73 ms; T_*I*_ 180 ms; T_*R*_ 12 s; in‐plane resolution 1.875 mm; matrix size 128^2^; flip angle 90°). Functional images, acquired with gradient EPI pulse sequence (T_2_*‐weighted), measured blood oxygen level‐dependent (BOLD) response. These consisted of 80 volumes of 16 near‐axial slices each (thickness: 7 mm; inter‐slice gap: 0.7 mm: T_*E*_ 40 ms; T_*R*_ 1600 ms; matrix size 64^2^; flip angle 90°; in‐plane voxel size 3.75 mm^2^).

### fMRI analysis

The software package *XBAM* version 4.1 (Centre for Neuroimaging Sciences, IOPPN at King's College London; www.brainmap.co.uk), was used to analyse the fMRI data. XBAM combines non‐parametric permutation‐based resampling methods with GLM statistics, goodness‐of‐fit ratios (SSQ), wavelet signal denoising methods, control of false‐positive voxels and clusters, and reports exact significances rather than results corrected for family‐wise error rates. Statistics and randomization procedures are described in the Appendix S1. All fRMI results are reported for ≤0.5 false‐positive rate.

### Comparison of time series and evoked haemodynamic responses

**Figure 1:**
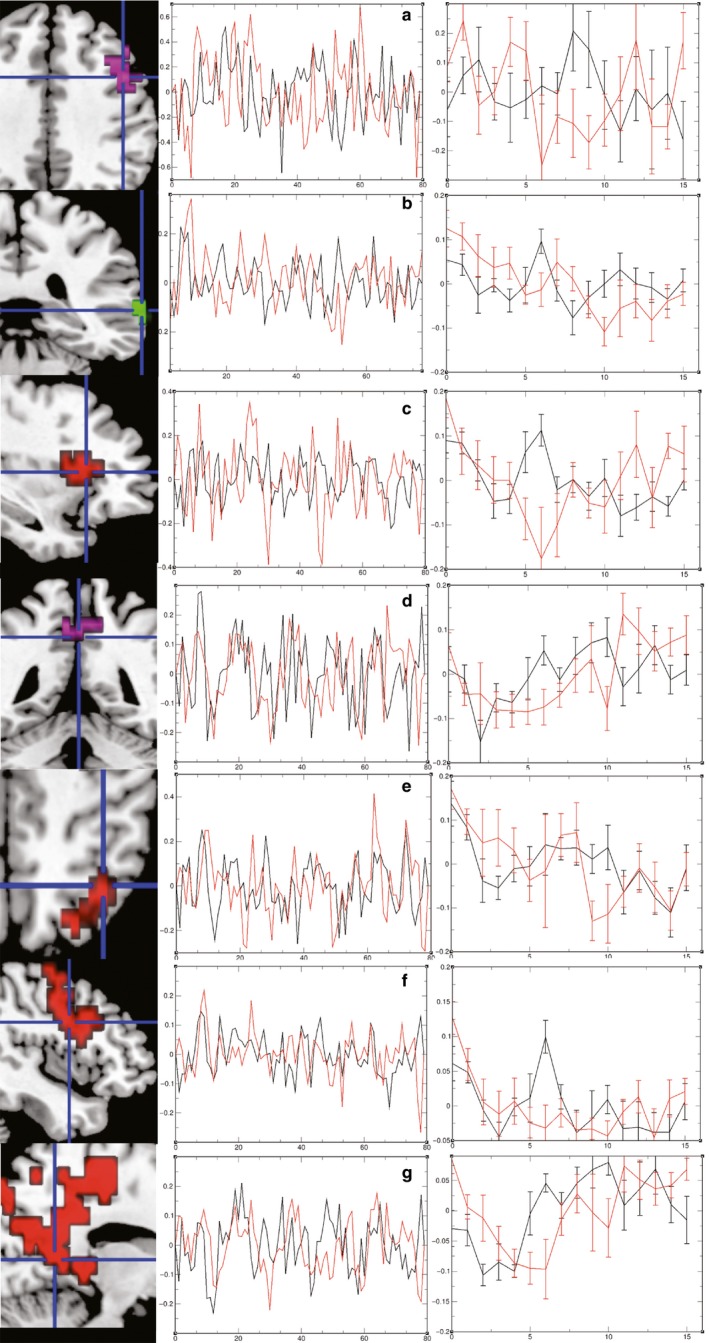
Basic activations and activation differences in time series and evoked haemodynamic responses. Radiological convention L* *= R. Colour codes in brain images refer to origin of cluster: red, group map non‐referred control (NC); green, group map depersonalization‐derealization disorder (DPRD); magenta, ancova image. Colour codes in time series plots: black, NC; red, DPRD. Left column: (a) inferior frontal sulcus; (b) supramarginal gyrus; (c) anterior insula/circular sulcus; (d) posterior cingulate gyrus BA31; (e) lateral precuneus; (f) middle frontal gyrus BA9; (g) lingual gyrus BA18. Middle column: plotted average time series. Right column: evoked haemodynamic responses.

Average BOLD signal time series were extracted from regions of interest (ROIs) based on group level and between‐group ancova images. An omnibus test for significance in overall differences implemented in XBAM 4.1 was used to compare randomized average time series at each time point using SSQs. Mean BOLD signal time series at each TR were used to plot evoked haemodynamic responses, enabling determination of group differences at each time point using confidence intervals (Fig. 1).

## Results

### Interference effect, NPE and response‐delay spans

Reaction time (RT) and response accuracy data were automatically recorded as described above. Percentages of correct responses were computed for each of the two conditions. SIEs were computed as between‐condition differences in RTs; differences in mean percentages correct responses (∆PC) were determined likewise. NPEs were computed for each block on a single trial‐by‐trial basis, as were ARDS, which were generated from subjects’ minima and maxima for each block in the active condition. Table 1 lists means and comparison of means in RT measures amongst the two experimental groups. The establishment of SIEs and NPEs in each group is reported in Appendix S1.

**Table 1:** Descriptives of Stroop experimental measures and psychophysiology

	NC		DPRD		*t*	*P*	95% CI	95% CI
	*M*	SD	*M*	SD				
Neutral condition RT	587.0	171.1	517.0	122.6	−1.092	ns	−280.0	−31.03
Negative condition RT	805.0	123.7	649.6	148.9	−2.615	0.015	−210.8	70.82
SIE	218.1	220.2	132.5	100.6	−1.190	ns	−251.5	80.49
PC neutral condition	97.81	3.335	94.44	3.340	−1.984	0.062	−0.136	0.016
PC negative condition	98.09	3.299	92.69	3.098	−2.287	0.034	−0.067	−0.002
ΔPC	0.288	3.653	−1.747	3.494	−0.748	ns	−7.732	3.552
NPE	21.09	37.44	28.17	31.63	−2.686	0.015	−0.405	−0.005
ARDS	72.13	35.07	105.7	60.62	2.428	0.048	0.087	1.045
Neutral condition ASCR	0.190	0.138	24.77	64.47	1.288	ns	−65.05	−15.89
Negative condition ASCR	0.292	0.139	0.509	0.305	2.087	0.052	−0.002	0.436
ΔASCR	0.359	0.028	0.521	0.549	0.880	ns	−0.223	0.548

ARDS, average response‐delay span; ASCR, amplitude of skin conductance response; CI, confidence interval; DPRD, depersonalization‐derealization disorder; NC, non‐referred control; NPE, negative priming effect; PC, percentage correct; RT, reaction time; SIE, Stroop interference effect. Behavioural response times in ms; electrodermal responses in μSiemens; log‐transformed scores used for *t*‐tests.

### Psychophysiology measurement and experimental consistency

**Figure 2:**
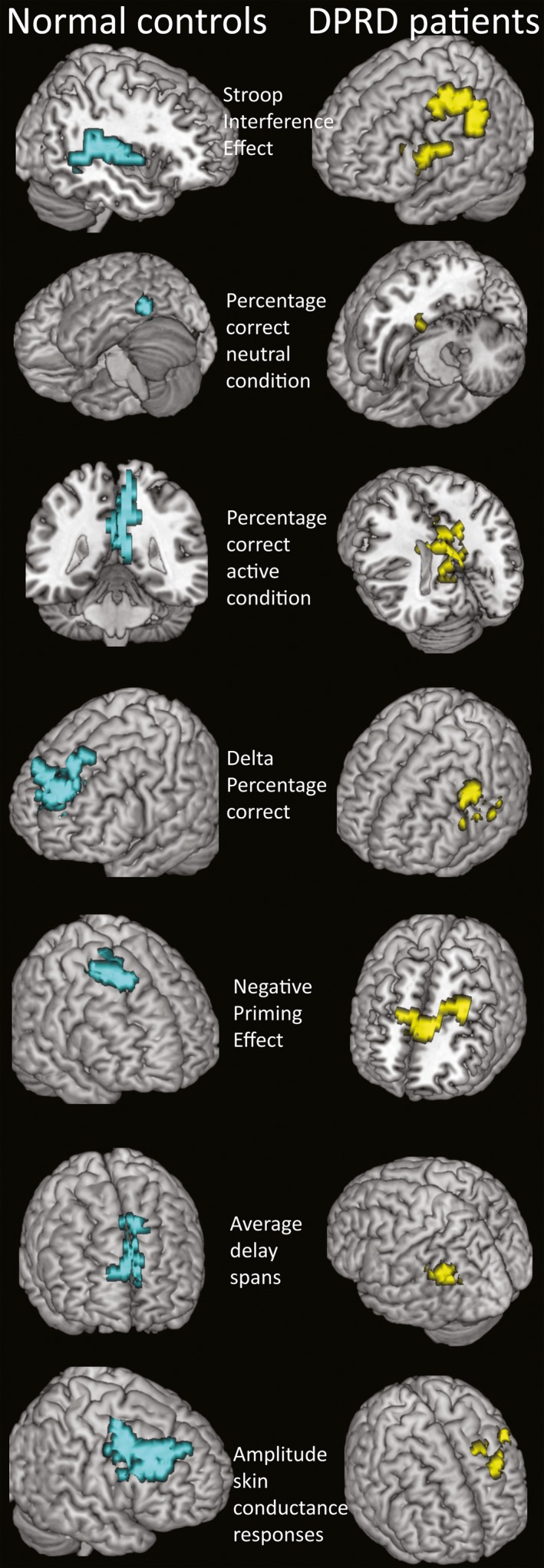
Main correlation clusters. Neurological convention R* *= R. Colour codes in brain images: turquoise, non‐referred control (NC); yellow, depersonalization‐derealization disorder (DPRD). For description of correlation clusters, see Table 3.

EDA data were filtered and processed in the way described above, and standard skin conductance variables were computed. Exploratory analyses had revealed that ASCRs exhibited the greatest sensitivity towards discrimination of the two groups, and also best experimental consistency. For reasons of clarity and brevity, therefore, this study focused solely on the ASCR variable out of all EDA measures. Table 2 lists between‐condition stability and discrimination tests for ASCR, RT measures, and responses misses and hits. These results suggest a high degree of consistency in the main experimental measures – both between‐condition correlations and *t*‐tests were significant (Fig. 2).

**Table 2:** Between‐condition stability and discrimination of main experimental measures

Measure	*r*	*P*	*t*	*P*	lower 95% CI	Upper 95% CI
ASCR	0.934	0.0001	−2.880	0.009	−0.220	−0.036
RT	0.591	0.005	−4.603	0.0001	99.21	263.6
No response	0.628	0.002	−1.848	0.080	−1.624	0.101
PC	0.702	0.0001	9.212	0.0001	2.984	3.821

ASCR, amplitude of skin conductance response; CI, confidence interval; RT, reaction time. Behavioural response times in ms; electrodermal responses in μSiemens; log‐transformed scores used for *t*‐tests.

### Group differences in behavioural and physiological data

As indicated in Table 1, there were no differences in neutral RT, but significant group differences in active incongruent condition RT. However, there was no significant difference in the SIE. The DPRD group had faster RTs in all comparisons. Regarding the percentages of correct responses, there was a significant group difference for the incongruent active condition, but none in the neutral condition. Again, ∆PC between‐group difference was not significant, although patients with DPRD had a lesser level of correct responses. Both the NPE and the ARDS variables were significantly larger for the DPRD group. These differences indicate that patients with DPRD may be more prone to distraction, consistent with them making fewer correct responses, and exhibited greater variability in response‐delays, the latter indicating lesser consistency in the active condition.

**Figure 3:**
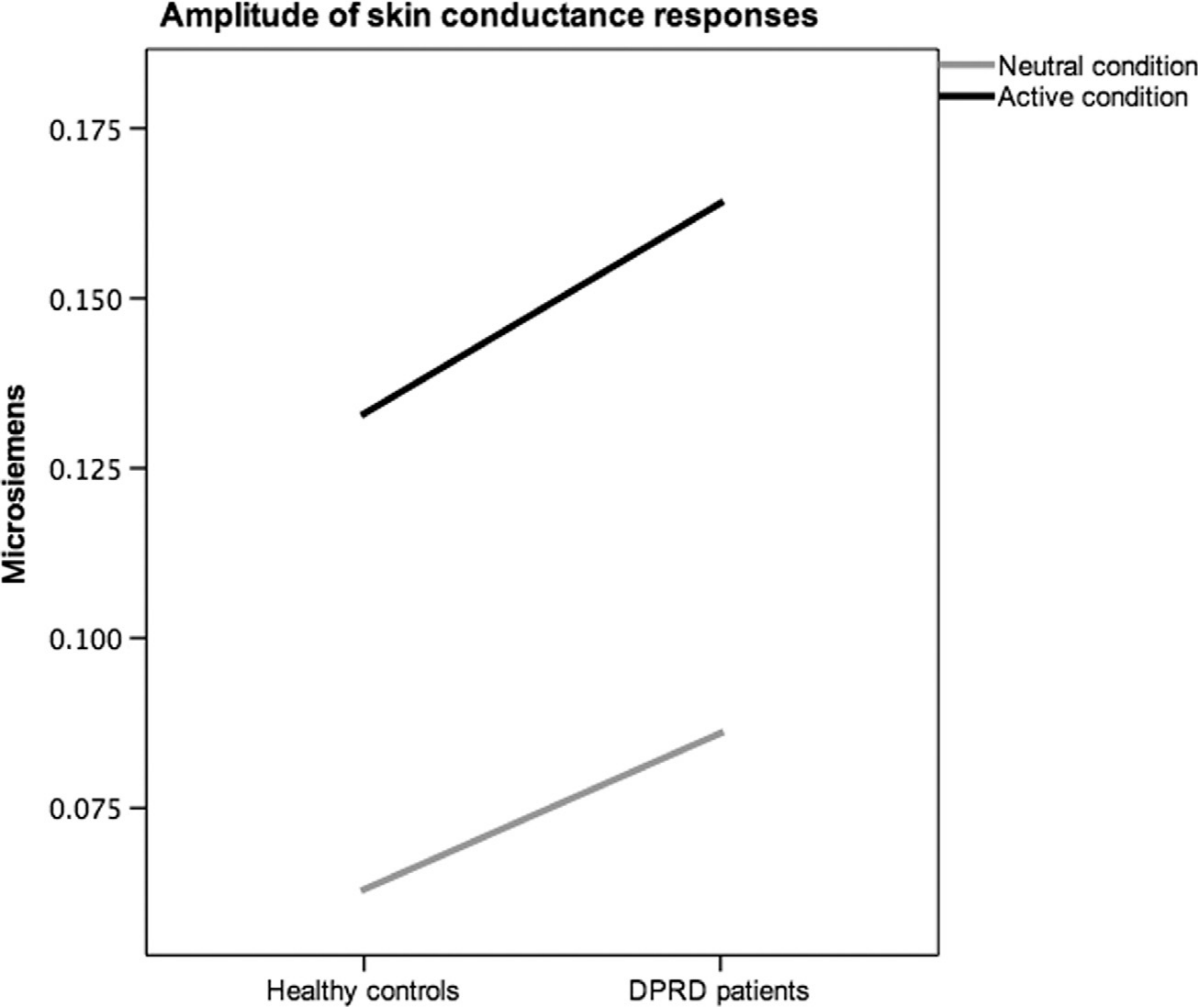
Amplitude of skin conductance responses (SCRs) across conditions. Amplitudes of SCRs averaged per epoch in μSiemens (mode) for each group in neutral and active conditions are plotted. The diagram visualizes differential arousal levels between groups, and relatively less effective arousal suppression in the depersonalization patient group.

Regarding electrodermal responses, averaged amplitudes of SCRs were larger in patients with DPRD (Fig. 3). These results reveal greater variability of sympathetic reactivity under cognitive task load for patients with DPRD, and relatively less effective arousal suppression in the active than in the neutral condition.

### Intercorrelations of main experimental measures within groups

Both experimental groups exhibited partly similar and partly disparate association patterns amongst the main experimental measures when controlling for confounding variables (Appendix S1; Table 3). In both groups, ASCRs correlate with ∆PC, PC_neut_, PC_neg_ and NPE; NPE and SIE. Reversal of association is present for correlations of ASCRs with ARDSs. These results may suggest that abnormal or ineffective sympathetic outflow could be at the core of inconsistencies in cognitive load performance of patients with DPRD. After application of Bonferroni‐adjustment of α‐levels, however, only the correlation NPE‐ASCR in the NC group, and NPE‐DPC and NPE‐SIE in the DPRD survive as truly significant.

**Table 3:** Group‐wise intercorrelations amongst main experimental measures

	*r*	*P*	
NC			
ΔPC	0.870	0.024	Negative PC
ΔPC	−0.899	0.015	Neutral PC
ΔPC	0.708	0.022	Skin conductance amplitude
Neutral PC	−0.908	0.009	Skin conductance amplitude
Negative PC	−0.605	0.048	Skin conductance amplitude
ARDS	−0.677	0.095	Skin conductance amplitude
NPE	−0.774	0.005*	Skin conductance amplitude
NPE	−0.748	0.013	Neutral PC
NPE	−0.798	0.006	Negative PC
SIE	−0.719	0.044	NPE
DPRD			
ΔPC	0.904	0.005*	Negative PC
ΔPC	−0.895	0.006	SIE
ΔPC	0.984	0.016	ARDS
Negative PC	−0.981	0.001*	SIE
Negative PC	−0.836	0.019	Skin conductance amplitude
Neutral PC	−0.873	0.054	Skin conductance amplitude
NPE	−0.991	0.006	Skin conductance amplitude
ARDS	0.875	0.023	Skin conductance amplitude
SIE	−0.988	0.009	ARDS
SIE	−0.746	0.021	NPE
SIE	0.842	0.017	Skin conductance amplitude

ARDS, average response‐delay span; DPRD, depersonalization‐derealization disorder; NC, non‐referred control; NPE, negative priming effect; PC, percentage correct; SIE, Stroop interference effect. Partial correlations controlling for date, daytime, state anxiety and alexithymia. *Significant at α** *= 0.005 following Bonferroni adjustment.

### Main activation and main correlation regions

It was possible to replicate the basic activation pattern found previously in the negative priming Stroop‐to‐neutral contrast (Steel *et al*., 2001; baseline subtracted, thresholds voxel level 0.05, cluster levels 0.004 and 0.003, respectively; Fig. 1): frontal (left precentral gyrus, inferior frontal sulcus, posterior wall, BA *BA*6, Talairach coordinates *XYZ* −36 0 26, cluster *P *= 0.000275), insular (right anterior insular gyrus, superior circular sulcus, 33 22 4, *P *= 0.000825) and parietal regions (right lateral precuneus, *BA*7, 33 −60 42, *P *= 0.002200), with deactivation in the occipital (right lingual gyrus *BA*18, 11 −52 4, *P *= 0.000278) lobe. The patients with DPRD showed ‘additional’ frontal (left middle frontal gyrus, middle frontal sulcus, *BA*9 −29 22 37, *P *= 0.000285) and parietal (left angular gyrus, *BA*39 −40 −63 37, *P *= 0.000285) activations, with deactivation in the dorsal posterior cingulate (*BA*31 4 −48 43, *P *= 0.000279). Hence, patients differed in the location (inferior vs. superior) of the parietal region, but were activating similarly in the left frontal region. The left dorsomedial prefrontal cortex *BA*9 (−30 23 37, *P *= 0.006686) and dorsal posterior cingulate cortex (*BA*31 4 −48 42, *P *= 0.003389) clusters reappeared in ancova images contrasting the groups as the main difference regions. The group maps of the active minus neutral conditions were the basis of correlation analysis with each of the experimental measures in each group. Table 4 summarizes the main correlation clusters in the two experimental groups. The two groups show substantially different correlation regions for the majority of the measures. Even with application of Bonferroni‐adjustment of α‐levels to α** *= 0.00714 per group, almost all correlation results remain significant.

**Table 4:** Correlation clusters of main NP Stroop experimental measures and psychophysiology

	NC							DPRD						
	Hemis	Size	*X*	*Y*	*Z*	*r*	*P*	Hemis	Size	*X*	*Y*	*Z*	*r*	*P*
SIE	R	110	54	37	2	0.63	0.002656	L	110	−47	7	48	0.92	0.003687
	*Precentral sulcus BA6/BA44*							*Middle frontal gyrus BA6*						
PC Neutral	L	28	−51	−48	−8	0.68	0.045768	L	53	−46	−44	−23	0.61	0.009501
	*Occipitotemporal cortex BA37*							*Fusiform gyrus/entorhinal cortex BA36*						
PC Negative	R	172	11	−52	15	0.89	0.002153	R	197	0	−44	26	0.97	0.001477
	*Posterior cingulate gyrus BA30*							*Posterior cingulate gyrus BA31*						
ΔPC	L	198	−7	63	26	0.91	0.001477	L	105	−54	33	20	0.58	0.003123
	*Superior frontal gyrus BA10*							*Middle frontal gyrus BA46*						
NPE	R	85	29	22	53	0.81	0.004676	L	111	−7	33	11	0.85	0.003940
	*Middle frontal gyrus BA6*							*Dorsal mid‐anterior cingulate gyrus BA24*						
ARDS	R	80	7	52	26	0.67	0.004629	L	41	−54	−52	31	0.49	0.011119
	*Superior frontal gyrus BA9*							*Supramarginal gyrus BA39*						
ASCR	R	189	43	22	37	0.96	0.003687	L	117	22	30	31	0.92	0.002744
	*Middle frontal gyrus BA9*							*Superior frontal gyrus BA9*						

ARDS, average response‐delay span; ASCR, amplitude of skin conductance response; DPRD, depersonalization‐derealization disorder; NC, non‐referred control; NPE, negative priming effect; PC, percentage correct; SIE, Stroop interference effect. Significance tested against 50 cycles of random permutation. Size, number of voxels in respective 3D cluster; *X*,* Y*,* Z* are the Talairach coordinates of the peak activation/correlation within the clusters.

### Functional connectivity

For each of the main clusters found in the correlation analyses, the BOLD signal levels were extracted from the individual cluster peaks in percentage effect size. To test for functional connectivity, these extracted values were tested for association (Table 5). Overall, functional connectivity (Table 5) correlation patterns partly replicate correlations also present in experimental measures (Table 3) in each of the groups. However, NC subjects show eight, and DPRD show only six significant paths. It may be concluded that patients with DPRD have slightly reduced functional connectivity. However, with application of Bonferroni‐adjustment of α‐levels, α** *= 0.00625 for NC, α** *= 0.00833 for patients with DPRD, only NPE‐ASCR and SIE‐NPE associations remain significant in the NC group; in the DPRD group, the two associations NPE‐ASCR and SIE‐ARDS survive. Thus, both groups would have an equal amount of connectivity.

**Table 5:** Functional connectivity of BOLD signal levels amongst main correlation regions

	*r*	*P*	
NC			
ΔPC	0.534	0.091	Negative PC
ΔPC	−0.595	0.054	Neutral PC
Negative PC	0.544	0.084	Neutral PC
Neutral PC	−0.615	0.044	Skin conductance amplitude
Negative PC	−0.605	0.048	Skin conductance amplitude
NPE	−0.774	0.005*	Skin conductance amplitude
SIE	−0.709	0.015	ARDS
SIE	−0.743	0.009	NPE
DPRD			
ΔPC	0.725	0.027	Negative PC
ΔPC	−0.716	0.030	SIE
Negative PC	−0.688	0.040	SIE
Negative PC	−0.833	0.005*	Skin conductance amplitude
SIE	−0.806	0.009*	ARDS
SIE	−0.746	0.021	NPE

ARDS, average response‐delay span; DPRD, depersonalization‐derealization disorder; NC, non‐referred control; NPE, negative priming effect; PC, percentage correct; SIE, Stroop interference effect. Bold signal percentage effect size extracted from individual peak voxels. *Significant at α** *= 0.006 (NC) and α** *= 0.008 (DPRD) following Bonferroni adjustment.

### BOLD time series and evoked haemodynamic responses

Visual inspection of time series extracted from ROIs for both groups (Fig. 1, middle column) suggests that patients with DPRD show a more extreme pattern of spiking. Testing for overall differences, however, reveals that there are no overall significant between‐group differences in average BOLD time series, except for a significant trend (*P *= 0.0961) in the supramarginal cluster. When inspecting the evoked haemodynamic responses (Fig. 1, right column), it can be observed that significant between‐group differences exist at between three and six out of 16 time points in the ROI clusters evaluated. These results suggest a partly distinct pattern of haemodynamic modulation in patients with DPRD.

## Discussion

This study used a Stroop word–colour interference task paradigm, combining a negative priming condition and a control condition, and investigated the neural correlates of the SIE, NPE, cognitive load, error rates and sympathetic stress responses. To this end, a group of DPRD and NC subjects were compared in behavioural, neural and autonomic measures. For basic activation markers, the groups were compared in terms of BOLD signal time series and evoked haemodynamic responses. For each experimental measure, the brain‐behaviour correlation regions in each of the two groups were computed. The interrelations of behavioural and psychophysiological measures were tested, and also functional connectivity patterns were tested for amongst the main brain‐correlation regions.

The basic results align to those of a preceding neuroimaging study establishing this negative priming Stroop paradigm (Steel *et al*., 2001), and confirm other activation patterns described in the literature (see below). The main behavioural findings of this study are that both groups exhibited SIEs and NPEs. The groups showed significant between‐group differences in NPE, cognitive load, specific error rates and skin conductance amplitudes. Substantially different locations of regions for brain‐behaviour correlations became evident for SIE, NPE and cognitive load. Such patterns of alternating and/or compensatory co‐activation actually resemble those typically seen in normal aging with cognitive tasks, whilst in the absence of severe brain pathology. When testing for functional connectivity, the DPRD group exhibited an overall reduced pattern of connectivity amongst the main correlation regions, but similar in interrelations when comparing with the NC group. Comparing experimental behavioural and psychophysiological interrelations, it became apparent that DPRD show an inverse association pattern of skin conductance with the cognitive load index measure, suggesting an abnormal mechanism of autonomic stress reactivity. When extracting BOLD time series from group maps, it became obvious that, whilst there are no significant overall group differences, the DPRD group show more extreme spiking patterns in BOLD responses. In evoked haemodynamic responses, significant differences in BOLD deflections were evident in 30–40% of time points compared.

Regarding testing of *a priori* hypotheses, the assumption of no group differences (hypothesis i) was not consistently supported. Indeed, both groups exhibited both SIEs and NPEs, but showed near‐significant between‐group differences in NPE, error rates, cognitive load and skin conductance amplitude (yet these trends did not persist after Bonferroni correction for multiple testing). These findings suggest that patients with DPRD exhibit lesser negative priming suppression, higher error rates, smaller working memory spans and less effective arousal suppression under stressful task demand. Regarding correlation regions for the SIE (hypothesis ii), activation in the inferior frontal junction and parietal regions was expected for NC. Confirming this hypothesis, NC subjects had their main correlation region in the right inferior frontal sulcus. For the NPE correlation regions, activations in superior, middle and inferior frontal gyri were expected (hypothesis ii), regions that had the largest BOLD effects in the Steel *et al*. (2001) study. The main correlation region for the NPE was in *BA*6, consistent with the hypothesis and literature (Derrfuss *et al*., 2004). For cognitive load correlation regions (hypothesis iii), activations were hypothesized where previously delay‐active neurons had been discovered: prefrontal, posterior parietal and striatal structures. Supporting this expectation, correlation regions for the ARDS were in the dorsomedial superior frontal cortex in NC subjects, and in the right supramarginal gyrus in patients with DPRD. For the ASCR, correlation regions were expected mainly in the SMA (hypothesis v), an expectation that received no clear support (as the clusters lie anterior to *BA*6, but may still be part of the SMA). It is possible that the orally based response modality in this task is responsible for the more lateral location, as compared with the manual output mode in the Zhang *et al*. (2012) study. However, DPRD showed mesial wall SMA involvement in ASCRs. With respect to the expectation of different locations in DPRD (hypothesis vi), specifically, in prefrontal memory regions due to possible stress alterations, this hypothesis could be supported for NPE, cognitive load and ASCRs, where patients with DPRD showed regions for brain‐behaviour correlations clearly distinct from those in NC subjects (Appendix S1 discussion).

This investigation has the advantage of utilizing a paradigm that can account for Stroop and NPEs, as well as for working memory span. Furthermore, the experimental setup as a multimodal imaging study allows considering findings that were previously dispersed in several methodological schools of thought. Although the relative rarity of primary DPRD diagnosis necessarily imposes restrictions on sample size, this study is based on countrywide sampling of patients. As the paradigm was a block design, an experimental limitation of the present study is that it was not possible to discern between correct‐only and incorrect‐only activations. This problem was, however, overcome by the use of brain‐behaviour correlation analyses.

In conclusion, it can be stated that the present investigation has yielded further evidence to support the notion that patients with DPRD do ‘not’ exhibit gross impairments in terms of selective attention, cognitive inhibition and working memory. However, slight neuropsychological deficits were shown and confirmed in terms of reduced short‐term memory, distractibility and inability to suppress stress‐related arousal states under cognitive task demand.

## Supporting information

Appendix S1. Methods.
