# Supplementary material for: Cognitive load and autonomic response patterns under negative priming demand in depersonalization‐derealization disorder
Source: Eur J Neurosci. 2016 Feb 15;43(7):971–8. doi: 10.1111/ejn.13183 (PMC4855951; doi:10.1111/ejn.13183)
Supplement: Supplementary file 1 — Appendix S1. Methods. [file EJN-43-971-s001.pdf]

Supplementary Online Materials

Supplemenatry Methods

Additional patient information

Six patients were diagnosed with minor co-morbid depression and/or anxiety disorder. Six patients were unmedicated, and three were receiving the lowest weaning doses of three different medications (including selective serotonin reuptake inhibitors, SSRIs, and neuroleptics: paroxetine, fluoxetine, olanzapine). Due to this circumstance, statistical control of medication effects were not feasible. The twelve NC subjects were chosen to match sample characteristics of DPRD patients. No specific differences in socio-demographic factors (education, SES) and gender ratio were found. Screening for achromatopsia and dyslexia was negative for all subjects.

Behavioural paradigm

Voice-activated software recorded subjects' responses and determined their content. Performance was then automatically evaluated in terms of reaction time (RT) and response accuracy (correct, missing and wrong responses) using custom software. The neutral condition has no discrepancy between hue colour and word content. This baseline condition comprised coloured Xs instead of written colour words in each trial — equal in character numbers to respective colour words, e.g. XXXXX in green hue. In the active probe condition, stimuli were single colour words (YELLOW, GREEN, RED, and BLUE) displayed in an incongruent font colour (e.g., "GREEN" displayed in red hue). Participants were instructed to ignore the word content and to name the font colour. In accordance with the classical Stroop task, a word-colour processing discrepancy was thus introduced by the naming of the hue colour of a visually presented colour word.

The deceleration of responses in the spelling of the colour name of the visual stimulus resulting from a discrepancy between stimulus colour and colour word content is termed the Stroop Interference Effect (SIE). The SIE is thus calculated as reaction time delay between neutral control and active (incongruent or negative) condition. The SIE has also been associated with the "central executive" component in working memory (Baddeley, 1996; Baddeley & Della Sala, 1996), presumably situated in the prefrontal cortex. In Negative Priming tasks, the preceding trial consists of a distracter stimulus (May *et al.*, 1995; MacLeod & MacDonald, 2000; Mayr & Buchner, 2007) presented before the target word. NPEs are considered to reflect inhibitory processes (Tipper, 2001; Mayr & Buchner, 2007) due to the exertion of cognitive control (de Fockert *et al.*, 2010). The NP Stroop paradigm requires subjects to respond to a target stimulus, which has previously been ignored as a distracter. The resulting change in reaction time is termed the Negative Priming Effect (NPE). For example, for the combined Stroop/Negative Priming stimuli, where hue and colour word were incongruent, the distracter (colour word) on trial n (prime) became the target (hue) on trial n+1 (probe) (e.g. "RED" written in blue hue (trial n) and "GREEN" written in red hue (trial n+1)). Reaction times for the NPEs were computed on a trial-by-trial basis and then averaged per block and per subject. As an index measure for cognitive load, we also examined the individual differences between minimal and maximal negative priming reaction times. We calculated the difference of these as the average delay spans (ARDSs) for negative priming delays per block. ARDSs could then be computed as the mean delays across the five active condition blocks. We assumed that ARDSs indicate the impact of cognitive loads (Sweller, 2003) reflected by working memory performance.

Stimulus presentation

The NP Stroop task was administered using a block design paradigm incorporating alternating neutral (control) and incongruent/Negative-Priming (active probe) conditions. The experimental run lasted 6:18 min in total (36 s per block). Each stimulus was presented on a black background (duration 100 ms), preceded by a fixation cross (duration 1500 ms) and followed by a blank screen (duration 2100 ms). Both neutral and active probe conditions were constructed so as to be identical in visual presentation. Stimuli were projected onto a screen that subjects could observe through the use of mirrors placed at 45° above their eyes. A compressed acquisition pulse sequence was used (Amaro *et al.*, 2002) to avoid image acquisition during overt responses (creating potential additional motion artefacts).

fMRI data analysis

Data were corrected for head movement by realignment to the average scan (Bullmore *et al.*, 1999). Linear low-frequency trends were removed, as well as high frequencies (low-pass filtering), correcting for baseline variations. Finally, after spatial smoothing with a 8.8 mm Full Width Half Maximum (FWHM) Gaussian filter, functional data were normalised onto each subject's own anatomical scan. Activation maps, expressing the neural correlates of brain activation during Stroop versus control task, were then computed independently for each individual subject. First, an activation model was fitted at each voxel by convolving the experimental design (active vs control blocks) with two Poisson functions (4 and 8 s), representing the haemodynamic response. These functions provide a scaling factor for every model component. The least-squares fit of the weighted sum of these two convolutions at every voxel produced a goodness-of-fit statistic termed sum of squares quotient (SSQ), which expresses the ratio of the model to

the sum of squares of its residuals. SSQ values can either be positive or negative, denoting activation or deactivation to the Stroop condition, respectively. The distribution of the SSQ statistic under the null hypothesis of no experimental effects (making no assumptions about the shape of the distribution) was computed by transformation of the time-series into the wavelet domain. The data were subsequently randomized 100 times, which effectively eliminated statistical power from the experimentally related components of the residuals (Bullmore *et al.*, 2001; Bullmore *et al.*, 2003). Activations for any contrast at any required *p*-value can then be determined by obtaining the appropriate critical values from the null distribution (generated from pulling together the randomised SSQs from all voxels in the analysis). Individual brain activation maps were produced for each subject for the Stroop vs. control condition.

All reported results are corrected for optimal rates of expected false-positives of ≤0.5 error 3D clusters over the whole brain volume (**Table 4**). We computed the Pearson product-moment correlation coefficient *r* between active task behavioural measures and blood oxygen level dependent (BOLD) effect data at each voxel. We then computed the null distribution of correlation coefficients by first permuting 50 times the active-task measures between the subjects and re-computing *r* for each data shuffle, before combining all the *r* values into one large null distribution. Cluster level maps were generated by combining adjacent voxels where *r* was found to be significant into 3D clusters and testing the cluster masses (sum of the *r* values over the 3D cluster) for significance against the masses of 3D clusters occurring by chance in the permuted data (the same process was used to produce cluster mass for the Stroop vs. control condition). BOLD signal level, in mean percentage effect size, was extracted from the peak voxel in the main clusters—individually ascertained for each single subject.

Supplementary Results

Establishment of Stroop Interference and Negative Priming Effects

Overall SIEs and NPEs were established by bootstrapped *t*-tests performed with 1000 iterations each (IBM SPSS 20 for Intel Mac, IBM Corp, Armonk, NY, U.S.A.). We found a significant SIE as mean difference between neutral and incongruent condition across groups (t=-4.043, df=17, P<0.001)(95%CIs -279.26- -87.73). We also observed a significant SIE in the NC group (t-3.057, df=10, P<0.012)(95%CIs -366.36- -57.43), as well as a significant SIE in the DPRD group (t=-3.204, df=6, P<0.019)(95%CIs -244.91- -32.79). There was a significant NPE as mean difference between minimal and maximal average delays in the incongruent condition across groups (t=-7,789, df=20, P<0.0001)(95%CIs -369.41- -213.34), a significant NPE in the NC group (t=-6,023, df=11, P<0.0001)(95%CIs -324.62- -150.87) and a significant NPE in the DPRD group (t=-5,607, df=8, P<0.001)(95%CIs -514.14- -213.63). Thus both groups exhibited the Stroop, as well as Negative Priming effects. Between-group differences are listed in **Table 1**.

Self-report measures

For control purposes, subjects completed the following self-report questionnaires: Spielberger's State-Trait Anxiety Inventory, Beck's Depression Inventory, Sierra's and Berrios' Cambridge Depersonalization Scale, Bernstein-Carlson's and Putnam's Dissociative Experience Scale, Rief's Complaint Somatization Inventory, Taylor's and Bagby's Toronto Alexithymia Scale. As expected, the two groups differed significantly on all of those scales (**Supplementary table 1**).

Blockwise examination of performance

ARDSs and NPEs allowed group performance for each trial block to be tested. Repeated univariate ANOVAs were chosen to examine the changes in reaction times over the duration of the experiment.

Within-subjects main effect for NPEs with Greenhouse-Geisser correction was F_{20}=2.695, *df*=2.707, *P*<0.047, partial-η^2=0.124. Contrast analysis revealed that the within-subjects effect was best represented by a cubic trend $F_{1,20}$=5.429, *P*<0.033, pη^2=0.218. The interaction NPE × group was significant, F_{20}=4.322, *df*=2.707, *P*<0.011, pη^2=0.185. This interaction was best fitted by a linear trend $F_{1,20}$=12.497, *P*<0.002, pη^2=0.397. Post-hoc *t*-tests confirmed one significant between-group contrast for block 5 at *t*=2.411, *df*=19, *P*=0.039 (CIs 0.091-2.852; logarithmised). NPEs were largest in the last block, and smallest in the first block: both groups performed better initially here, but reduced NPEs over the duration of the experiment.

Within-subjects main effect for ARDS with Greenhouse-Geisser correction was F_{20}=13.584, *df*=2.831, *P*<0.0001, pη^2=0.417. Contrast analysis revealed that this within-subjects effect was best represented by a linear trend $F_{1,20}$=26.782, *P*<0.0001, pη^2=0.585. The interaction time × group was also significant, F_{20}=3.699, *df*=2.831, *P*<0.019, pη^2=0.163. This interaction was best fitted by a cubic trend $F_{1,20}$=5.696, *P*<0.028, pη^2=0.231. Post-hoc *t*-tests confirmed one significant between-group contrast at *t*=2.627, *df*=19, *P*=0.017 (CIs 0.185-1.636; logarithmised) for block 1. Response delay spans were largest initially, with both groups narrowing spans during the course of the experiment, thereby linearily reducing ARDSs. This narrowing over time can be interpreted as a training process towards better efficacy in short-term memory. By application of Bonferroni-adjustment of α-levels at α*=0.025, however, only group

differences for the ARDS should be considered conservative enough to reject the null hypothesis.

Examination of experimental data for confounds

Reaction time and psychophysiological data were examined for possible confounding factors. Age, sex, and education were found unrelated (*r*s<0.3) to the experimental measures. Daytime and scan dates were observed to show moderate covariation (*r*s>0.3) with SIE, NPE, ARDSs, ΔPC and ASCRs. Of self-reports, the most systematic moderate (*r*s>0.3) confounders were severity of alexithymia and state anxiety levels. State anxiety is well-known in the literature on Stroop experiments, alexithymia is also well-known to reflect upon autonomic functioning. These confounding variables were therefore controlled for in partial correlations whenever appropriate. To examine potential bias for one hue colour, reaction times were also investigated for colour effects. The mean RTs of the four colours showed stability by intercorrelations (*r*s 0.695~0.976, all *p*s<0.0001). Pairwise t-tests and ANOVAs showed no between-group effects. Word lengths showed intercorrelations (*r*s 0.854~0.984, all *p*s<0.0001). Pairwise t-tests and ANOVAs confirmed that no between-group effects exist. These results also support consistencies for colours and word lengths, and exclude the possibility of response biases amongst groups due to stimulus properties.

Connectivity with primary symptom measures

Using an exploratory approach, we tested if any of the BOLD signal levels extracted from the main correlation regions **(Table 4)** would show association with any subscales of the CDS, indicating a relation to depersonalisation symptoms. We adjusted Hierarchical Regression Analysis models (robust Ordinary Least Squares regression, STATA 12.1 MP,

StataCorp, College Station, TX, USA) to include all main association regions and the set of confounders previously identified. Beforehand analyses using Multivariate Regression had indeed demonstrated a multivariate compound effect of confounders (F=7.086, P=0.041) also for the occurrence of depersonalisation states. In normal controls, ARDS correlation region superior frontal gyrus (*BA*9) showed a significant regression slope with depersonalisation frequency (t=48.87, P=0.0001; CIs 0.0045-0.0053), when adjusted for differential clustering in education levels. In DPRD patients, ARDS correlation region supramarginal gyrus (*BA*39) exhibited a significant regression slope with depersonalisation frequency (t=-61.82, P=0.004; CIs -9.183-7.846), after adjustment for differential clustering between education levels. These findings suggest that in both experimental groups, frequency of depersonalisation states are in relation with a measure of capacity for cognitive load, influenced by trait anxiety, emotional illiteracy, and dependent on education levels. Because there is no difference between the two groups in this mechanism, it is rather unlikely to have clinical implications as such, although the DPRD group engage a remarkably different region for the same cognitive function.

Supplementary Reference Section

Amaro, E., Jr., Williams, S.C., Shergill, S.S., Fu, C.H., MacSweeney, M., Picchioni, M.M., Brammer, M.J. & McGuire, P.K. (2002) Acoustic noise and functional magnetic resonance imaging. *J Magn Reson Imaging*, **16**, 497-510.

Baddeley, A. (1996) The fractionation of working memory. *Proc Natl Acad Sci U S A*, **93**, 13468-13472.

Baddeley, A. & Della Sala, S. (1996) Working memory and executive control. *Philos Trans R Soc Lond B Biol Sci*, **351**, 1397-1403; discussion 1403-1394.

Bullmore, E., Fadili, J., Breakspear, M., Salvador, R., Suckling, J. & Brammer, M. (2003) Wavelets and statistical analysis of functional magnetic resonance images of the human brain. *Stat Methods Med Res*, **12**, 375-399.

Bullmore, E., Long, C., Suckling, J., Fadili, J., Calvert, G., Zelaya, F., Carpenter, T.A. & Brammer, M. (2001) Colored noise and computational inference in neurophysiological (fMRI) time series analysis: resampling methods in time and wavelet domains. *Hum Brain Mapp*, **12**, 61-78.

Bullmore, E.T., Brammer, M.J., Rabe-Hesketh, S., Curtis, V.A., Morris, R.G., Williams, S.C., Sharma, T. & McGuire, P.K. (1999) Methods for diagnosis and treatment of stimulus-correlated motion in generic brain activation studies using fMRI. *Hum Brain Mapp*, **7**, 38-48.

de Fockert, J.W., Mizon, G.A. & D'Ubaldo, M. (2010) No negative priming without cognitive control. *J Exp Psychol Hum Percept Perform*, **36**, 1333-1341.

MacLeod, C.M. & MacDonald, P.A. (2000) Interdimensional interference in the Stroop effect: uncovering the cognitive and neural anatomy of attention. *Trends Cogn Sci*, **4**, 383-391.

May, C.P., Kane, M.J. & Hasher, L. (1995) Determinants of negative priming. *Psychol Bull*, **118**, 35-54.

Mayr, S. & Buchner, A. (2007) Negative priming as a memory phenomenon: a review of 20 years of negative priming research. *Zeitschrift für Psychologie*, **215**, 35-51.

Sweller, J. (2003) Evolution of human cognitive architecture. *The Psychology of Learning and Motivation*, **43**, 215-266.

Tipper, S.P. (2001) Does negative priming reflect inhibitory mechanisms? A review and integration of conflicting views. *Q J Exp Psychol A*, **54**, 321-343.

Supplementary Figure 1 ANCOVA profile plots

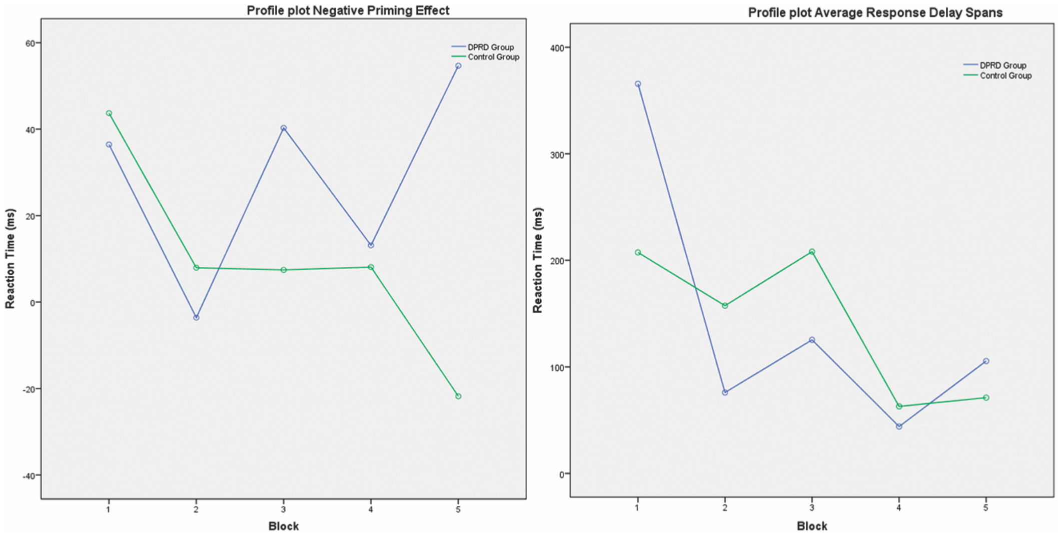

Supplementary Table 1 Self-report measures

Instruments and scales	Controls *M*	*S.E.M.*	Depersonalisation *M*	*S.E.M.*	Student's *t*	*p* Value
Edinburgh Handedness Right	15.083	0.9908	14.889	2.3478	0.084	ns
Edinburgh Handedness Left	1.750	0.7398	2.777	1.3517	-0.713	ns
Dissociative Exp Scale Tot Sc	185.83	44.830	518.89	120.81	-2.870	0.010
Dissociative Exp Scale Mean Sc	6.2198	1.5841	17.583	4.6012	-2.608	0.017
SOMS-2 Complaint Somat Index	1.4167	0.5961	10.111	3.2843	-2.994	0.007
TAS-20 Mean Score	36.666	3.6041	52.444	4.2068	-2.853	0.010
TAS-20 Factor I	1.6035	0.1253	4.4603	1.7302	-1.914	0.071
TAS-20 Factor II	2.4308	0.2826	4.0277	0.9641	-1.793	0.089
TAS-20 Factor III	2.1320	0.1547	4.9166	2.1410	-1.508	ns
FBCS Total Score	221.91	10.357	234.66	6.3617	-0.965	ns
Cambridge Depers Scale Durat	0.3248	0.0723	1.3984	0.2455	-4.730	0.000
Cambridge Depers Scale Freq	0.5105	0.1021	2.7057	0.5303	-3.922	0.004
Cambridge Depers Scale Global	26.667	4.7821	87.889	18.476	-3.643	0.002
Cambridge Depers Scale Total	53.333	9.5642	175.77	36.952	-3.643	0.002
Cambridge Depers Scale State M	3.6060	1.7177	30.141	5.8063	-4.382	0.002
Beck Depression Inventory	3.5830	1.3951	17.444	2.9302	-4.291	0.001
Spielberger State STAI-Y1	1.8292	0.1371	2.438	0.2875	-2.075	0.052
Spielberger Trait STAI-Y2	2.0208	0.1285	2.472	0.2666	-2.383	0.028

Note:—df=19. Unequal between-group variances were assumed in computation of *t*-tests. 210 words
